# Improving quality of life through the routine use of the patient concerns inventory for head and neck cancer patients: baseline results in a cluster preference randomised controlled trial

**DOI:** 10.1007/s00405-020-06077-6

**Published:** 2020-06-01

**Authors:** Simon N. Rogers, Christine Allmark, Fazilet Bekiroglu, Rhiannon Tudor Edwards, Gillon Fabbroni, Robert Flavel, Victoria Highet, Michael W. S. Ho, Gerald M. Humphris, Terry M. Jones, Owais Khattak, Jeffrey Lancaster, Christopher Loh, Derek Lowe, Cher Lowies, Dominic Macareavy, James Moor, T. K. Ong, A. Prasai, Nicholas Roland, Cherith Semple, Llinos Haf Spencer, Sank Tandon, Steven J. Thomas, Andrew Schache, Richard J. Shaw, Anastasios Kanatas

**Affiliations:** 1 Faculty of Health and Social Care, Edge Hill University, Ormskirk, L39 4QP UK; 2 Leeds Teaching Hospitals and St James Institute of Oncology, Leeds Dental Institute and Leeds General Infirmary, Leeds, UK; 3 Liverpool Head and Neck Centre, Liverpool University Hospital Aintree, Liverpool, UK; 4 Centre for Health Economics and Medicines Evaluation (CHEME), School of Healthcare Sciences, College of Health and Behavioural Sciences (CoHaBS), Ardudwy Building, Normal Site, Bangor University, Bangor, UK; 5 Guildford, UK; 6 Liverpool Head and Neck Clinical Trials, Clinical Sciences Building, University Hospital Aintree, Liverpool, UK; 7 School of Medicine, Medical and Biological Sciences, North Haugh, St Andrews, UK; 8 Liverpool Head and Neck Centre, University of Liverpool, Cancer Research Centre, 200 London Road, Liverpool, L3 9GA UK; 9 Astraglobe Ltd, Congleton, Cheshire UK; 10 Institute of Nursing and Health Research, Ulster University, Shore Road, Newtownabbey, Co. Antrim, BT37 0QB UK; 11 South Eastern Health and Social Care Upper Newtownards Road, Belfast, BT16 1RH UK; 12 Oral and Maxillofacial Surgery Department, Bristol University, Lower Maudlin Street, Bristol, UK

**Keywords:** Head and neck cancer, Intervention, Prompt list, Health-related quality of life, Randomised trial, Patient concerns inventory, Cluster preference

## Abstract

**Purpose:**

The main aim of this paper is to present baseline demographic and clinical characteristics and HRQOL in the two groups of the Patient Concerns Inventory (PCI) trial. The baseline PCI data will also be described.

**Methods:**

This is a pragmatic cluster preference randomised control trial with 15 consultant clusters from two sites either ‘using' (*n* = 8) or ‘not using’ (*n* = 7) the PCI at a clinic for all of their trial patients. The PCI is a 56-item prompt list that helps patients raise concerns that otherwise might be missed. Eligibility was head and neck cancer patients treated with curative intent (all sites, stage of disease, treatments).

**Results:**

From 511 patients first identified as eligible when screening for the multi-disciplinary tumour board meetings, 288 attended a first routine outpatient baseline study clinic after completion of their treatment, median (IQR) of 103 (71–162) days. At baseline, the two trial groups were similar in demographic and clinical characteristics as well as in HRQOL measures apart from differences in tumour location, tumour staging and mode of treatment. These exceptions were cluster (consultant) related to Maxillofacial and ENT consultants seeing different types of cases. Consultation times were similar, with PCI group times taking about 1 min longer on average (95% CL for the difference between means was from − 0.7 to + 2.2 min).

**Conclusion:**

Using the PCI in routine post-treatment head and neck cancer clinics do not elongate consultations. Recruitment has finished but 12-month follow-up is still ongoing.

## Introduction

Following head and neck cancer (HNC) patients can experience substantial physical, emotional, and social dysfunction as post-treatment consequences effects many aspects with a detrimental impact on health-related quality of life (HRQOL) [1, 2]. These in turn can lead to a significant burden of unmet supportive care needs [3]. There is evidence that given the opportunity patients value the chance to discuss their concerns [4]. There are various tools available to assist the clinician in the identification of potential unmet needs in the HNC setting, of which the Patient Concerns Inventory (PCI) is one. In a systematic review, Shunmugasundaram et al. recommended the PCI, particularly when considering the importance of content validity over quantitative psychometric properties [5]. The PCI is a condition-specific item prompt list [6] and is based on the literature around the use of questionnaire prompt lists in consultations [7]. It consists of 56 items, which patients select from before their appointment, to help guide the outpatient consultation, which covers a range of symptoms and potential problems individuals may confront after treatment. The PCI helps to focus the consultation, to aid doctor-patient communication, and to signpost patients to other professionals for advice and support [8]. It is a tool that can be integrated into routine clinical practice [9].

Although pilot work has confirmed that most patients wish to continue to use the PCI in their consultations [10], and that its inclusion alters the items discussed with clinicians and potentially improves HRQOL [11, 12], there is a lack of evidence from a randomised trial of patient benefit, which has prompted this novel trial [13]. By conducting a pragmatic, cluster-based, multi-centre randomised controlled trial (C-RCT) involving several consultants, it will be possible to evaluate with rigor, whether the repeated inclusion of the PCI in routine post-treatment consultations does make a significant and clinically meaningful difference in patient-reported HRQOL and distress.

The main aim of this paper is to present baseline demographic and clinical characteristics and HRQOL in the two groups of the Patient Concerns Inventory (PCI) trial. Also, to describe the baseline PCI data. The baseline data provides the opportunity to describe HRQOL and patient concerns relatively early on in the post-treatment trajectory. Having a greater understanding of early post-treatment issues and challenges could help shape interventions at this critical time of adaptation to the cancer diagnosis and the consequences of treatment.

## Material and methods

The study methods have been described previously [13]. Briefly, this is a pragmatic cluster-controlled trial, with consultants (clusters) randomised to ‘using’ or ‘not using’ an intervention incorporating the PCI prompt list at all their trial clinics. Individual patient randomisation was ruled out because of the likely sensitization of consultants to using the PCI. Such sensitization could lead to certain strategies being carried over to when control group patients were being seen, and consequently a possible dilution of any intervention effect. It is also a consultant preference trial, by which those with preferences were given their preferred group and those with no preference were randomised. This was to limit the chance of PCI-sceptic consultants dominating the PCI group and PCI-enthusiastic consultants the non-PCI group. The allocation process was overseen by the trial medical statistician, before any patient recruitment occurred. Two centres participated. In Leeds, three of six consultants preferred to be in the PCI group, while the three others had no preference and were placed in the control group. In Liverpool, three of eight consultants preferred to be controls and the other five had no preference: one was randomised to the control group and the other four were placed in the PCI intervention group. One newly appointed consultant at Liverpool joined the trial soon after it started and was randomised to the PCI group.

Sample size calculations required 312 patients from ten or more consultants to show (with 80% power, 5% level of significance) a halving in the percentage with less than the good overall quality of life at the final follow-up clinic, i.e. at 1-year on from the (baseline) first routine outpatient clinic after completion of treatment. With an expected 25% loss through attrition and non-consent, 416 was the required number to be identified at Multidisciplinary Team (MDT) Meetings. Eligible patients were given an information sheet about the trial and if willing they were asked to sign a consent form when they next attended hospital. Patients consented to their clinical data being used and to completing research questionnaires before each post-treatment consultation, some of which could be used in their consultation. Neither consultants nor patients were blind to the randomisation; this was a pragmatic trial. Eligible patients were treated curatively for primary HNC, and all sites, stages of disease and treatments were included. Patients with a second primary tumour were also accepted from January 2018. Patients treated palliatively and patients with a recurrence were excluded, as were patients with a history of cognitive impairment, psychoses or dementia. Eligible patients who later started palliative care discontinued their participation. Unit Clinical Trials Nurses recruited patients and dedicated funded Unit researchers collected baseline clinical and demographic data using a baseline clinic questionnaire based on that of the Head and Neck 5000 project [14], or by extraction from baseline clinical records. Index of Multiple Deprivation (IMD 2019) scores were derived from patient postcodes using publicly available data [15] and these provide a relative measure of deprivation at a small area level across England. Trial Quality Assurance included initial training and immediate post-consultation feedback from PCI patients about how much use had been made of the PCI prompt list.

**Fig. 1:**
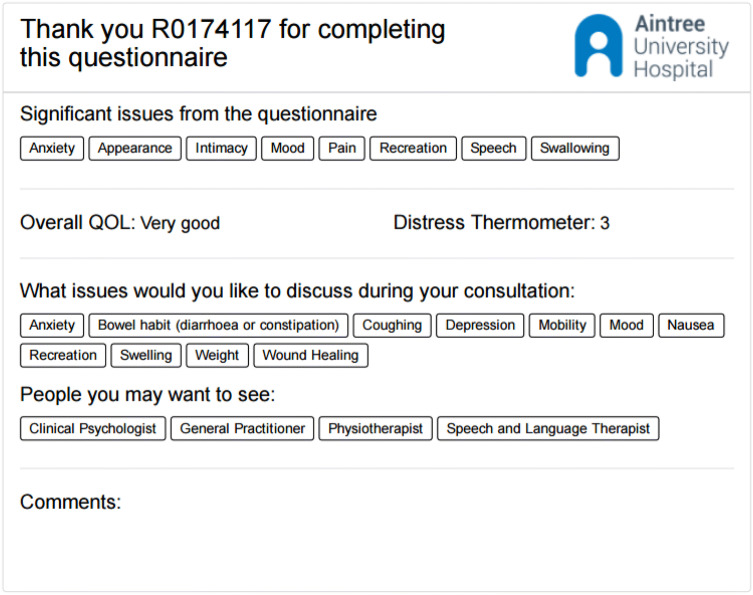
Example of a summary sheet that PCI patients took into their consultation

Pre-consultation questionnaires including the PCI prompt list were completed electronically (desktop, tablet, iPAD) apart from at one Liverpool hospital (non-PCI consultant) which used paper because of technical issues. All PCI intervention group patients were then given a sheet of paper summarising their data which they took into their consultations (Fig. 1). This sheet listed (a) all the PCI items they had selected for discussion, (b) any University of Washington (UWQOL) questionnaire domains in which a significant problem or dysfunction was indicated, (c) their overall UWQOL response and (d) their Distress Thermometer score. This summary sheet was the visible difference between the trial arms as far as the contact between consultant and patient was concerned. Control patients completed exactly the same pre-clinic information as intervention patients apart from the PCI prompt list, but crucially both they and their consultant did not see any summary sheet.

### Measures

The UW-QOL v4 questionnaire consists of 12 single-item domains, these having between 3 and 5 response options scaled evenly from 0 (worst) to 100 (best) according to response hierarchy [16]. UW-QOL domains are presented within two subscales, physical function and social-emotional function [17]. The physical function score is the mean of the appearance, swallowing, chewing, speech, taste and saliva domain scores, while the social-emotional function score is the mean of the pain, activity, recreation, shoulder, mood, and anxiety domain scores. Criteria derived from earlier work can be used to indicate the domains in which patients have a significant problem or dysfunction [18]. Question domains for intimacy and fears of recurrence were also developed using a similar system of possible responses as the UWQOL v4 [19, 20]. There is also a single item overall QOL question on the UWQOL v4 for which patients are asked to consider not only physical and mental health, but also other factors, such as family, friends, spirituality or personal leisure activities important to their enjoyment of life. The trial primary outcome measure is the percentage of patients with less than good overall QOL at the final 1-year clinic. As inference will target the individual patient level, the final analyses will include the adjustment for consultant clustering as well as for baseline variables including the baseline value of the QOL measure. Secondary outcomes include the mean social-emotional subscale score of the UWQOL-v4 and a Distress Thermometer (DT) score of 4 or more [21]. These will be reported once the trial has finished. HRQOL data also included the EQ-5D-5L [22].

### Statistical analysis

For cluster randomised trials the risk of chance imbalance is greater than for individual patient randomisation because of the relatively small number of clusters involved. It is therefore prudent to present relevant summary baseline information for both clusters (consultants) and patients. Fishers Exact (for categorical variables) and the Mann–Whitney test (for numerical variables) were used at the individual level to compare the case-mix of the two groups.

## Results

### Recruitment process and participation rate

**Fig. 2:**
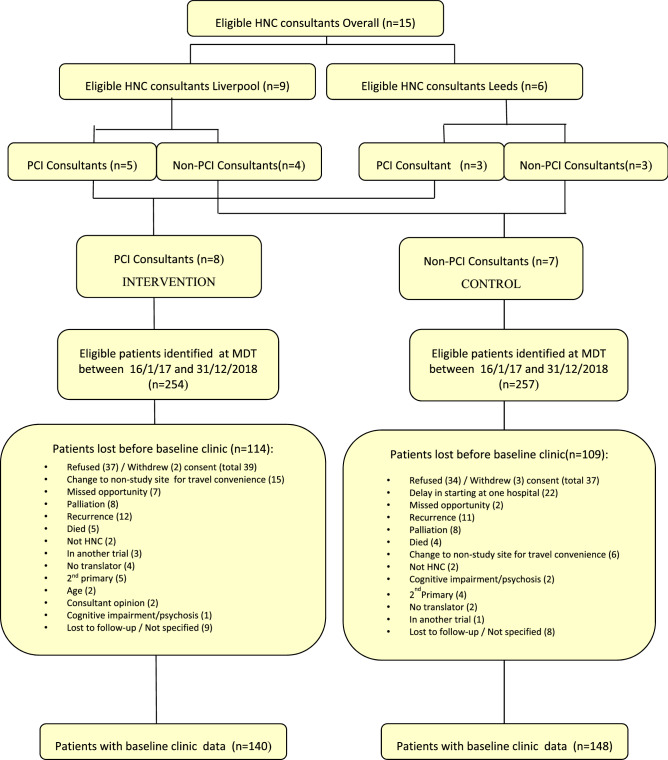
Patient flow diagram

Patients recruited to the trial and having baseline data were first discussed at MDT meetings between January 2017 and December 2018, with baseline trial clinics between April 2017 and October 2019. MDT meetings identified 511 potentially eligible patients (Fig. 2), with 288 attending baseline clinics a median (IQR) of 194 (125–249) days after diagnosis, and 103 (71–162) days after the end of treatment. The 223 patients not starting the trial included 111 who could not possibly have started it because of what materialised in the time lag between MDT and the trial baseline clinic to make them ineligible; these comprised 48 lost through death, tumour recurrence or palliation, 21 lost because it was more convenient for some patients to travel to a non-trial site, 22 because of delays in trying to agree an electronic system at one hospital and 20 for clinical reasons (in another trial, mental health, not HNC, 2nd Primary). The other 112 patients lost to the trial comprised 71 due to consent being refused, five who withdrew consent, nine by missed opportunity to recruit within a busy outpatient clinic environment, six when there was no translator available, four with exclusions outside the trial protocol (clinical opinion, patient age) and 17 lost to follow-up/unknown status. All 15 eligible consultants from the two locations took part in the trial, with a median (range) of 16 (5–48) patients attending baseline clinics.

### Randomisation baseline comparisons

At the cluster (consultant) level there was a similarity between trial groups in the number of patients per consultant, median consultation times, mean patient ages, and experience (Table 1). There was also a similarity in regard to the primary outcome variable as measured at baseline, i.e. the percentage with less than the good overall quality of life, as well as for key secondary measures, namely the mean social-emotional subscale score of the UWQOL and the percentage of patients reporting a distress thermometer score of four or more. However, there were differences between trial groups in the type of patient (tumour location and staging) seen by consultants and in treatments administered. PCI group consultants had fewer patients with oral cavity tumours, more with advanced tumour staging, and more receiving radiotherapy and chemotherapy. These differences in consultant case-mix transferred through to patient-level (Table 2), though less markedly reflecting the way the variation in patients per consultant has played out in the aggregation of data. Apart from these differences the two trial groups were similar in demographic and clinical characteristics. Of particular note is that consultation times were very similar, with PCI group times taking about 1 min longer on average (95% confidence interval for the difference between means was from 0.7 of a minute less to 2.2 min longer). There was also a similarity in regard to the primary outcome variable as measured at baseline, as well as for the key secondary measures, and also across a wide range of baseline QOL measures (Table 3).

**Table 1:** Baseline comparisons at the cluster (consultant) level

		PCI group	Control group
Consultant level			
No of consultants (patients)		8 (140)	7 (148)
No of patients in trial		Median: 16 Range: 9, 15, 15, 16, 16, 18, 23, 28	Median: 17 Range: 5, 6, 13, 17, 26, 33, 48
Year started as a consultant		Median: 2009 Range: **97**, 01, 06, 07, 10, 14, 17, 17	Median: 2009 Range: 03, 05, 09, **09**, **13**, 15, 15
Specialty	ENT or MFU	5 ENT, 3 MFU	2 ENT, 5 MFU
Consultation times (minutes)	Median	Median: 11 9, 10, 10, 11, 11, **11**, 13, 15	Median: 11 Range: 7, **8**, 10, 11, 11, 12, **12**
% of patients having	Surgery	Median: 30 Range: **0**, 13, 25, 27, 32, 52, 60, 72	Median: 58 Range: 17, **40**, 41, 58, 58, 62, **67**
	RT or RT/CT	Median: 38 Range: 0, 0, 7, 38, 38, 43, 47, **67**	Median: 6 Range: **0**, 0, 0, 6, 8, 31, **40**
	Surgery & (RT or RT/CT)	Median: 33 Range: 25, 27, 28, **33**, 33, 38, 48, 50	Median: 38 Range: **20**, **33**, 35, 38, 42, 52, 53
% of patients with	Advanced 3–4 staging	Median: 69 Range: 33, 39, 47, 68, 69, 73, 81, **89**	Median: 50 Range: 31, 31, 39, **50**, **60**, 65, 79
% of patients with tumour location	Oral cavity	Median: 15 Range: 0, 0, 4, 7, **22**, 80, 89, 100	Median: 77 Range: **0**, 2, 77, 77, 82, 85, **100**
	Oropharynx	Median: 37 Range: 0, 6, 7, **33**, 40, 46, 56, 56	Median: 15 Range:** 0**, 8, 9, 15, 18,** 60**, 73
	Larynx	Median: 22 Range: 0, 0, 7, **11**, 32, 38, 40, 44	Median: 0 Range:** 0**, 0, 0, 0, 0, 19, **40**
	Other	Median: 7 Range: 0, 0, 6, 6, 7, 13, 18, **33**	Median: 6 Range: **0**, **0**, 0, 6, 6, 8, 15
Patient age at baseline	Median	Median: 63 Range: 58, 61, **61**, 62, 63, 63, 64, 65	Median: 61 Range: **57**, 57, 59, 61, 62, **63**, 68
Patient gender	% Female	Median: 32 Range: 13, 13, 21, 31, **33**, 44, 47, 70	Median:31 Range: **0**, 13, 24, 31, 39, 45, **50**
UWQOL Overall Quality of life	% Less than good	Median: 34 Range: **22**, 22, 25, 32, 35, 38, 40, 40	Median: 27 Range: **0**, 23, 24, 27, **33**, 38, 42
UWQOL social-emotional subscale	Median	Median: 78 Range: **65**, 70, 76, 78, 78, 83, 83, 85	Median: 78 Range: 63, 67, 69, 78, **78**, 83, **85**
Distress thermometer (DT)	% ≥ 4	Median: 56 Range: 30, 32, 33, **56**, 56, 60, 63, 67	Median: 54 Range: 29, 31, 31, 54, 55, **60**, **67**

The results bold are those of the three consultants with fewer than ten study patients. These results are liable to greater fluctuation

**Table 2:** Baseline case-mix comparisons at the patient level

		PCI group *N* = 140	Control group *N* = 148	*P* value
Hospital	Aintree	59% (82)	65% (96)	0.28
	Leeds	41% (58)	35% (52)	
Days from diagnosis to baseline clinic	Median (IQR)	189 (120–255)	195 (125–244)	0.68
Days from end of treatment to baseline clinic	Median (IQR)	108 (70–165)	102 (75–160)	0.90
Duration of consultation (minutes)	Mean, Median (IQR)	11.9, 11 (8–15),* n* = 137	11.1, 10 (7–13),* n* = 147	0.09
Gender	Female	35% (49)	28% (41)	0.20
Age at baseline	Median (IQR)	63 (57–69)	60 (53–68)	0.12
Ethnicity	A1 (White British)	99% (138)	95% (141)	0.17
Tumour site	Oral cavity	39% (55)	53% (79)	0.002
	Oropharynx	30% (42)	33% (49)	
	Larynx	21% (30)	7% (11)	
	Other	9% (13)	6% (9)	
Clinical T stage	3–4	21% (30)	24% (36)	0.58
Clinical N stage	Positive	54% (75)	44% (65)	0.13
Overall stage	0/I	26% (36)	32% (48)	0.47
	II	14% (20)	14% (20)	
	III	20% (28)	14% (21)	
	IV	40% (56)	40% (59)	
Primary treatment	Surgery only	38% (53)	43% (63)	0.02
	RT only	9% (13)	2% (3)	
	RT&CT only	18% (25)	11% (17)	
	Surgery and RT	27% (38)	36% (54)	
	Surgery and RT and CT	8% (11)	7% (11)	
Free-flap transfer	YES	25% (25/102)	33% (42/128)	0.19
WHO comorbidity	0	63% (88)	61% (91)	0.28
	1	20% (28)	26% (39)	
	2–4	17% (24)	12% (18)	
ACE27 comorbidity	None	51% (71)	45% (66)	0.52
	Mild	29% (41)	36% (54)	
	Moderate	18% (25)	16% (23)	
	Severe	2% (3)	3% (5)	
Living situation	Alone in house/flat	21% (29)	25% (36/146)	0.48
Working	Yes	36% (48/134)	27% (40/146)	0.16
Financial benefits	Yes	39% (49/127)	42% (58/138)	0.62
Smoking habit	Current	12% (16/135)	14% (21/145)	0.77
	Former	60% (81/135)	57% (82/145)	
	Never	28% (38/135)	29% (42/145)	
Alcohol habit	Current	74% (100/135)	65% (94/145)	0.25
	Former	22% (30/135)	30% (43/145)	
	Never	4% (5/135)	6% (8/145)	
IMD 2019 quintile	1 = least deprived	11% (16)	12% (18)	0.83
	2	21% (29)	18% (26)	
	3	16% (22)	18% (27)	
	4	12% (17)	16% (23)	
	5 = most deprived	40% (56)	36% (54)

**Table 3:** Baseline clinic QOL comparisons at the patient level

		PCI group	Control group
		*N* = 140	*N* = 148
Main outcome measures			
UWQOL Overall Quality of life	Less than good	32% (45)	30% (44)
UWQOL social-emotional subscale	Mean, Median (IQR)	75, 78 (64–87)	70, 72 (55–88)
Distress thermometer (DT)	Score ≥ 4	47% (66)	43% (63)
Other measures			
UWQOL Overall Quality of life	Outstanding	8% (11)	5% (7)
	Very good	26% (36)	34% (51)
	Good	34% (48)	31% (46)
	Fair	24% (34)	20% (29)
	Poor	4% (5)	8% (12)
	Very Poor	4% (6)	2% (3)
UWQOL physical function subscale	Mean, Median (IQR)	69, 69 (57–86)	67, 68 (53–86)
UWQOL items			
Social-emotional subscale			
Pain	Best possible response	38% (53)	37% (55)
	Dysfunction	28% (39)	30% (44)
Activity	Best possible response	33% (46)	27% (40)
	Dysfunction	10% (14)	14% (20)
Recreation	Best possible response	44% (61)	32% (48)
	Dysfunction	6% (8)	10% (15)
Shoulder	Best possible response	61% (85)	51% (75)
	Dysfunction	10% (14)	15% (22)
Mood	Best possible response	34% (48)	33% (49)
	Dysfunction	12% (17)	19% (28)
Anxiety	Best possible response	34% (47)	38% (56)
	Dysfunction	17% (24)	17% (25)
Physical function subscale			
Appearance	Best possible response	31% (43)	22% (32)
	Dysfunction	8% (11)	11% (17)
Swallowing	Best possible response	36% (50)	36% (54)
	Dysfunction	12% (17)	17% (25)
Chewing	Best possible response	41% (57)	41% (60)
	Dysfunction	14% (19)	13% (19)
Speech	Best possible response	46% (64)	40% (59)
	Dysfunction	7% (10)	9% (13)
Taste	Best possible response	29% (41)	33% (49)
	Dysfunction	19% (27)	20% (30)
Saliva	Best possible response	29% (41)	26% (39)
	Dysfunction	37% (52)	32% (47)
Other items			
Intimacy	Best possible response	79% (110)	69% (102)
	Dysfunction	4% (6)	6% (9)
Fear of recurrence	Best possible response	14% (19)	16% (24)
	Dysfunction	25% (35)	27% (40)
EQ-5D			
Mobility	Moderate/severe/unable	18% (25)	27% (40)
Self-care	Moderate/severe/unable	11% (16)	11% (16)
Usual activities	Moderate/severe/unable	21% (29)	28% (42)
Pain	Moderate/severe/extreme	31% (44)	30% (44)
Anxiety/depression	Moderate/severe/extreme	11% (15)	22% (33)
EQ-5D-5L TTO crosswalk values	Mean, Median (IQR)	0.76, 0.77 (0.68–0.88)	0.70, 0.74 (0.58–0.88)
Visual analogue scale (VAS)	Mean, Median (IQR)	73, 75 (60–87)	71, 76 (59–85)

### Baseline study population characteristics

Median (IQR) age at baseline clinic of the 288 patients was 62 (55–69) years and 69% (198) were male. For 47% (134) the tumour was located in the oral cavity, 32% (91) oropharynx, 14% (41) larynx and 8% (22) in other places. All but six tumours were squamous cell (SCC). Clinical staging was early (0–2) for 43% (124) and advanced (3–4) for 57% (164). Treatment for 40% (116) comprised surgery alone while 6% (16) received radiotherapy without surgery, 15% (42) radiotherapy and chemotherapy without surgery, 32% (92) surgery with adjuvant radiotherapy and 8% (22) surgery with adjuvant radiotherapy and chemotherapy. Free-flap transfer occurred for 29% (67/230) of operations, and specifically for 46% (60/130) of tumours in the oral cavity, 8% (5/61) oropharynx, 4% (1/23) larynx, and 6% (1/16) other locations. WHO comorbidities were at level 1 for 23% (67), level 2 for 13% (36), levels 3–4 for 2% (6). ACE 27 comorbidities were mild for 33% (95), moderate for 17% (48), and severe for 3% (8). Most patients had smoked with 13% (37/280) currently smoking at the time of the baseline questionnaire, 58% (163/280) being former smokers and 29% (80/280) having never smoked. Two-thirds (69%, 194/280) continued to drink alcohol, 26% (73/280) no longer did so and 5% (13/280) had never drank alcohol. One quarter (23%, 65/286), lived alone in a house or flat, 31% (88/280) were still working in paid employment, and 40% (107/265) were in receipt of financial benefits. Some 38% (110/288) of patients lived in an area that is ranked nationally (England) within the most deprived 20% of areas, according to overall deprivation scores (IMD 2019).

Overall QOL was outstanding for 6% (18), very good for 30% (87), good for 33% (94), fair for 22% (63), poor for 6% (17) and very poor for 3% (9); i.e. 31% (89) had overall QOL that was less than good and 69% (199) had overall QOL that was good or better. Also, 45% (129) stated Distress Thermometer values of ≥ 4 and the median (IQR) UWQOL emotional subscale score was 75 (59–88). Dysfunction in regard to UWQOL items were notably higher in regard to salivation (34%, 99), pain (29%, 83), fears of recurrence (26%, 75), and taste (20%, 57).

**Fig. 3:**
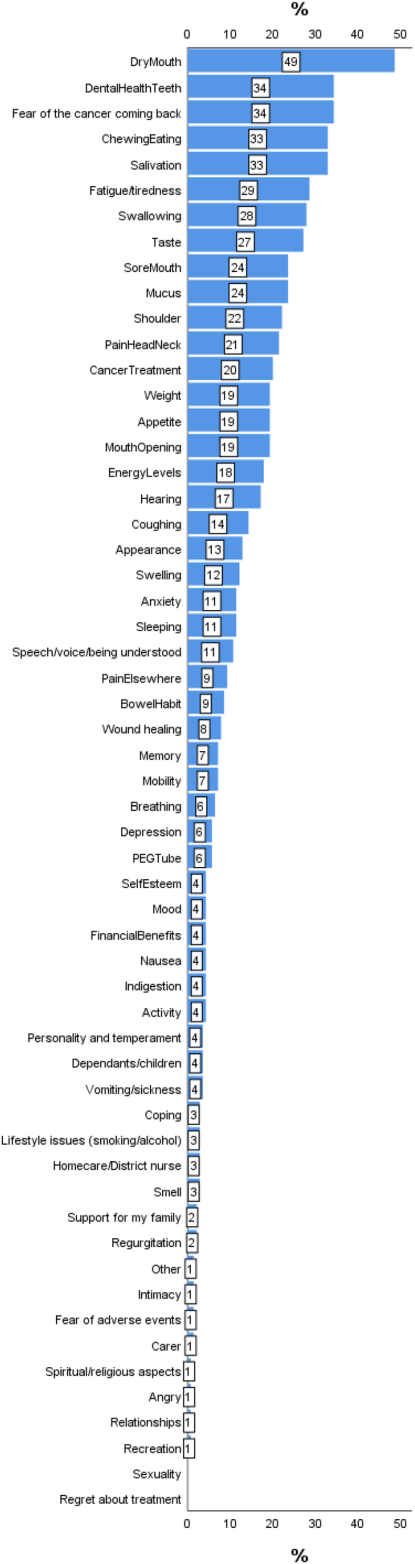
Graphic–bar chart of PCI frequencies

The mean number of items selected by 140 PCI patients for discussion in their consultation was 6.60, median (IQR) 5 (2–9), range 0–28 with 15 or more items selected by 9% (13) (Table 4). Nearly half (48%) of patients selected ‘dry mouth’ to discuss and other items selected by more than 20% were ‘fear of cancer coming back’ (34%), ‘dental health/teeth’ (34%), ‘chewing/eating’ (33%), ‘salivation’ (33%), ‘fatigue/tiredness’ (29%), ‘swallowing’ (28%), ‘taste’ 27%, ‘sore mouth’ (24%), ‘mucus’ (24%), ‘shoulder’ (22%), ‘pain in the head and neck’ (21%) and ‘cancer treatment’ (20%), (Table 4, Fig. 3). Almost all the immediate post-consultation feedback was positive in that the PCI had been used ‘a great deal’ or ‘somewhat’ in the consultation. The PCI results were broadly similar for the two sites involved in the trial.

**Table 4:** Patient PCI data at the post-treatment baseline clinic

		Aintree	Leeds	Combined
		*N* = 82	*N* = 58	i = 140
No of PCI selected: overall	Mean, Median (IQR)	6.91, 5 (2–10)	6.16, 5 (3–8)	6.60, 5 (2–9)
PCI items selected by domain				
No of PCI selected: Physical function	Mean, Median (IQR)	5.28, 4 (2–8)	4.76, 5 (2–7)	5.06, 4 (2–7)
No of PCI selected: cancer treatment	Mean, Median (IQR)	0.35, 0 (0–1)	0.31, 0 (0–1)	0.34, 0 (0–1)
No of PCI selected: social	Mean, Median (IQR)	0.39, 0 (0–1)	0.16, 0 (0–0)	0.29, 0 (0–0)
No of PCI selected: psychological	Mean, Median (IQR)	0.89, 1 (0–1)	0.93, 1 (0–1)	0.91, 1 (0–1)
PCI items selected by at least 20% of patients	Dry mouth	48% (39)	50% (29)	49% (68)
	Fear of cancer coming back	34% (28)	34% (20)	34% (48)
	Dental health/Teeth	30% (25)	40% (23)	34% (48)
	Chewing/eating	34% (28)	31% (18)	33% (46)
	Salivation	33% (27)	33% (19)	33% (46)
	Fatigue/tiredness	27% (22)	31% (18)	29% (40)
	Swallowing	32% (26)	22% (13)	28% (39)
	Taste	28% (23)	26% (15)	27% (38)
	Sore mouth	30% (25)	14% (8)	24% (33)
	Mucus	21% (17)	28% (16)	24% (33)
	Shoulder	22% (18)	22% (13)	22% (31)
	Pain in the head and neck	18% (15)	26% (15)	21% (30)
	Cancer treatment	20% (16)	21% (12)	20% (28)
No. of Health professionals selected	Mean, Median (IQR)	1.13, 1 (0–2)	0.48, 0 (0–1)	0.86, 0 (0–1)
Post consultation feedback on the PCI				
Did the doctor make reference to the PCI prompt list during the consultation?	Not at all	–	–	–
	A little	–	3	3
	Somewhat	1	12	13
	A great deal	80	37	117
	unknown	1	6	7

## Discussion

The main focus of this paper is to assess the baseline balance in this trial. Ivers et al. comment that many cluster randomised trials (C-RCTs) have too few clusters to reasonably expect cluster level balance [23]. They cite a review of C-RCTs with a median of 21 clusters per trial with 25% having fewer than 12 clusters. Our trial recruited 15 consultant clusters and we have presented data at both cluster and patient levels. The two trial groups were broadly similar at the patient level in demographic and clinical characteristics and over a wide range of QOL measures, apart from differences in regard to tumour location, tumour staging, and the mode of treatment. These exceptions are cluster (consultant) related to Maxillofacial and ENT consultants seeing different types of cases. Baseline imbalances are unimportant if the variable concerned is unrelated to the outcome, while strong predictors of outcome are important even if statistically non-significant [24]. It is important to identify strong baseline predictors and to adjust for these in the final analyses [25]. Our trial protocol named age, gender, treatment, overall clinical stage, and tumour site as case-mix variables that will be adjusted for. As the outcome data is awaited the strongest predictors are yet to be identified. We will also adjust for the baseline value of the QOL outcome so as to adjust for any imbalances between PCI and control groups.

A 1-year unfunded extension to the trial was necessary for various reasons. Firstly, the MDT identified potential patients in a wide catch-all net so as not to miss eligible patients but in so doing this process flagged patients who on closer scrutiny could never have been included had the consenting process taken place at the first post-treatment routine clinical outpatient review. This applied to 50% of those lost to the trial between MDT and trial baseline clinic. Secondly there were delays in setting up the IT capacity (iPAD) at a spoke unit and after several months trying to resolve this issue it was decided to allow paper completion because this involved only one non-PCI consultant. Vigilance of the clinical trials team at this site enabled robust data completion. IT difficulties do raise a wider issue around the future rollout of the PCI approach in HNC consultations across the NHS and currently a cloud-based platform is being explored. A third reason for extending the trial period was a long time lag to the baseline clinic than originally expected. The first baseline surgeon consultant review was at around 6 months following diagnosis, with a trial follow-up period of another year. Even with this trial extension to allow more patients to be recruited, the final outcomes will be analysed on fewer numbers than planned, probably on around two-thirds of the intended number. In hindsight an extra trial hospital would have been useful to the trial but we have to be pragmatic and in the final analyses accept that “size and power are irrelevant once the experiment has actually been carried out. At this point the trial is analysed using confidence intervals to show the plausible values for the treatment effects” [26].

The choice of a cluster design is a strength of the study because randomisation of consultants avoided contamination and possible dilution of results by consultants being sensitized with the PCI process. An additional robustness to the trial is the relatively small proportion of refusals and withdrawals, though this was anticipated given the very high participation levels for using the PCI in routine non-trial settings [4, 9, 10]. The number missed for logistical reasons was also small. Good patient and consultant recruitment allow confidence in respect of representativeness and acceptance of the PCI approach into clinical practice. Another strength of the trial was the electronic data capture which has virtually eliminated missing data in the outpatient clinic setting apart from the trial baseline questionnaires which patients could complete as they wished and from post-consultation feedback information of PCI patients.

The baseline assessment reflects survivors at a median of 6 months after diagnosis and 3 months after the end of treatment and their clinical characteristics are typical of a HNC patient population. HRQOL data highlights the dysfunction relating to salivation/dry mouth both in the UWQOL and as the most commonly raised issue on the PCI. There was also notable dysfunction in regard to pain and in fearing their cancer would return, and these issues were also commonly raised on the PCI. Whereas just over two-thirds of patients regarded their overall QOL as being good or better, distress levels were relatively high with nearly half having Distress Thermometer scores of four or more.

Consultation times were similar between the two groups indicating that the use of the PCI did not appear to have impacted the timetabling of clinic sessions. There is evidence around the benefit of questionnaire prompt lists [7]and how its use re-directs the focus of the consultation time onto those most pertinent issues for each patient [27]. Furthermore, from the issues raised on the PCI, this allows the HN team to apportion the necessary type and level of healthcare and supportive interventions, hence a more efficient use of time and personalized approach to routine follow-up care [27]. The PCI consultations were only about 1 min longer than non-PCI consultations and this should have no bearing on delivery across the NHS. There is a good indication from the immediate post-consultation questionnaires that the PCI was being used ‘a great deal’ at baseline and this will also be tracked over later clinics. One of the main criticisms that has been raised about the PCI is the possibility that by allowing patients to raise a lot of issues this will extend the consultation time unduly. The median number of PCI items selected was five, with an inter-quartile range of 2–9, which implies that the vast majority of patients do have issues they want to talk about and sometimes they have a lot of issues with one in ten patients having selected 15 or more items at these trial baseline clinics. This raises the issue of prioritisation within the consultation and anecdotal feedback from PCI consultant training sessions would suggest that they are able to incorporate items identified seamlessly into their consultations. Previous taped consultations have revealed that the time spent on physical examination in the consultation room, such as palpation of the neck, mouth examination and nasendoscopy takes just a few minutes [28]. As nasendoscopy takes longer this might account for the consultation time difference between ENT and MFU. In addition, it is possible to have relatively short consultations as these are set in the context of ongoing intervention from a range of professionals such as Clinical Nurse Specialist, Speech and Language Therapist, Dietitian, and Physiotherapists. Patients use the opportunity outside of the consultation, either by phone, email, or face to face, to access their support if this is not explicitly triggered during the clinic visit.

As the trial is set within the context of NHS practice and involved 15 consultants across two centres, the findings should have generalisability. Recruiting clusters is often more difficult than recruiting individuals [23], however, in this trial all eligible consultants agreed to participate. The choice of a cluster design is a strength of the study because randomisation of consultants avoided contamination and possible dilution of results by consultants being sensitized with the PCI process. Patients were allocated to individual consultants through the cancer tracking referral process and there was no selection bias based on knowing which consultants were using PCI and which were not. This has been a source of contamination in other trials [29]. Anecdotally, there was very little discussion observed between trial patients by the trial staff in the waiting room prior to consultations, which minimises any possible dilution of any effects of the intervention.

## Conclusion

The PCI trial is well balanced at baseline. Trial follow-up will continue until September 2020.
